# Aspects of In Vitro Biodegradation of Hybrid Fibrin–Collagen Scaffolds

**DOI:** 10.3390/polym13203470

**Published:** 2021-10-10

**Authors:** Marfa N. Egorikhina, Irina I. Bronnikova, Yulia P. Rubtsova, Irina N. Charykova, Marina L. Bugrova, Daria D. Linkova, Diana Ya. Aleynik

**Affiliations:** Federal State Budgetary Educational Institution of Higher Education, Privolzhsky Research Medical University of the Ministry of Health of the Russian Federation, 603005 Nizhny Novgorod, Russia; ira-bronikova-2014@yandex.ru (I.I.B.); rubincherry@yandex.ru (Y.P.R.); irina-ch0709@yandex.ru (I.N.C.); marysmir@mail.ru (M.L.B.); linckovadaria@yandex.ru (D.D.L.); daleynik@yandex.ru (D.Y.A.)

**Keywords:** scaffold, biopolymers, mesenchymal stem cells, collagen, fibrinogen, biodegradation, structure, biomedical cell product, hydrogel

## Abstract

The success of the regenerative process resulting from the implantation of a scaffold or a tissue-engineered structure into damaged tissues depends on a series of factors, including, crucially, the biodegradability of the implanted materials. The selection of a scaffold with appropriate biodegradation characteristics allows for synchronization of the degradation of the construct with the processes involved in new tissue formation. Thus, it is extremely important to characterize the biodegradation properties of potential scaffold materials at the stage of in vitro studies. We have analyzed the biodegradation of hybrid fibrin–collagen scaffolds in both PBS solution and in trypsin solution and this has enabled us to describe the processes of both their passive and enzymatic degradation. It was found that the specific origin of the collagen used to form part of the hybrid scaffolds could have a significant effect on the nature of the biodegradation process. It was also established, during comparative studies of acellular scaffolds and scaffolds containing stem cells, that the cells, too, make a significant contribution to changes in the biodegradation and structural properties of such scaffolds. The study results also provided evidence indicating the dependency between the pre-cultivation period for the cellular scaffolds and the speed and extent of their subsequent biodegradation. Our discussion of results includes an attempt to explain the mechanisms of the changes found. We hope that the said results will make a significant contribution to the understanding of the processes affecting the differences in the biodegradation properties of hybrid, biopolymer, and hydrogel scaffolds.

## 1. Introduction

Tissue engineering based on scaffold technology is an especially dynamic field in the development of regenerative medicine. Scaffolds are three-dimensional matrices that mimic natural extracellular matrices and provide temporary support for cells [1]. Scaffolds can be colonized by cells in vitro and then used for the transfer of these cells to an implantation site. They themselves can also act as biologically active materials that ensure cell recruitment from the neighboring tissues after implantation in vivo [2,3,4]. Thus, the main objective for scaffolds post-implantation is to provide appropriate conditions for the regeneration and successful repair of tissues in the damaged area. This objective is best reached through a combination of specific characteristics of such scaffolds: no cytotoxicity, biocompatibility, low immunogenicity, and a certain degree of mechanical strength, porosity, etc. [5,6,7].

One of the determinants for the design or selection of a scaffold is its biodegradability. Biodegradation is a process of the gradual destruction of the material of the scaffold, mediated by the biological activity of the environment into which the scaffold is placed. Biodegradation is an important property for biomaterials used as temporary scaffolds and/or implants. The relevance of studying such properties of scaffolds is confirmed by the rapidly growing interest of the scientific community in this field. An analysis of a publications cluster (publications) dated from 1990–2019, conducted by A.A.M. Shimojo et al. (2020), clearly showed an increase in the number of works related to studies both of the degradation of scaffolds in vivo or in experimental models mimicking natural conditions. For instance, in works dated from 2010–2019, “biodegradability” (or “biodegradation”) as a keyword was encountered eight times more frequently than in works dated from 1990 to 1999 [8].

The biodegradability of a scaffold allows it to be broken down over a defined period of time, giving way to the newly formed tissue [9,10]. It is important to consider the scaffold degradation rate specifically for each tissue type. Inappropriate scaffold selection can lead to clinical complications. For instance, in the case of a too-fast scaffold degradation, cells will end up unsupported, which could affect their functional activity and viability. By contrast, an overly slow biodegradation rate can lead to chronic inflammation and to scarring in the future. Thus, the process of scaffold degradation should be synchronous with its replacement with new tissue. That is, a scaffold’s degradation rate should be compatible with the rate of new tissue formation [11,12].

The degradation of scaffolds in the body can occur as a result of physical (dissolution), chemical (hydrolysis), and biological processes (enzymatic degradation or interaction with cells of the immune system). Enzymatic activity is most likely to be the largest contributor to the biodegradation of scaffolds after implantation. It is known that enzymes act as biological catalysts, greatly accelerating chemical reactions. Serine proteases (trypsin, plasmin, etc.) and matrix metalloproteinases (collagenases, etc.) are the types of enzymes that most often populate lesions [13,14]. These enzymes actively destroy the damaged tissue within the lesion, cleaning it of protein degradation products while allowing new tissue formation. In this regard, one can expect that these enzymes will actively interact with the scaffold as their substrate, resulting in its biodegradation [15,16,17]. The mechanism of the enzymatic biodegradation of scaffolds is mediated by a number of actions: enzyme diffusion from the bulk solution through the substrate matrix, enzyme adsorption onto the substrate followed by the formation of an enzyme–substrate complex, hydrolysis accompanied by a number of catalytic reactions, and the diffusion of soluble decomposition products from the substrate into solution [18]. As a result of such a breakdown, there will be losses in scaffold volume and mass, these losses providing evidence of its biodegradation [19]. There are various techniques to simulate the biodegradation process in vitro. A range of solutions can mirror the effects of natural body fluids, for example, phosphate buffer solutions, cell culture media, or enzyme solutions are used for this purpose. There are many ways to assess biodegradation in vitro, but there are two particularly common approaches. The first is based on determining the concentration of scaffold components released into the medium in which the scaffold is being incubated and degraded. Generally, this approach is used for scaffolds having proteins as their structure-forming components [20,21]. The second approach is characterized by examining the residual mass of the scaffold after incubation in the body-fluid-like solution. This approach is the more commonly applied of the two as it can be used for most types of scaffolds [22,23].

It should be noted that the rate and extent of scaffold biodegradation depends primarily on the material used to form the basis of its construction. All materials can be divided into natural and synthetic origin. Natural materials such as collagen, fibrin, hyaluronic acid, etc., are characterized by high biological activity and good biocompatibility. However, their biodegradation rate often exceeds that required, resulting in the structure degrading faster than is appropriate to support the regeneration [24,25]. The following modifications of scaffolds are used to regulate their biodegradation rate: crosslinking with chemical agents or by exposure to UV radiation, the inclusion of enzyme inhibitors in the scaffold structure, treatment with various substances that increase the resistance of the scaffold material to biodegradation, etc. [26,27,28,29,30].

Synthetic materials (polycaprolactone, polyglycolide, polyurethane, etc.), in general, are more durable and robust [31]. However, their degradation products can change the pH of the medium or are toxic to cells, leading to inflammation and to the disruption of regeneration [32,33,34]. The absence of biological activity is another problem with synthetic materials. In this regard, the field of development of composite scaffolds that combine natural and synthetic materials has started to develop rapidly. Composite scaffolds can combine the advantages of natural and synthetic materials, providing products with desired characteristics, including a range of different biodegradation properties [35,36].

In addition to the nature of the material chosen as the basis of scaffold formation, biodegradation may depend on the pH, the availability of functional groups, scaffold architectonics, and other parameters [37]. For example, porous scaffolds degrade faster than those with no or very small pores. This is the consequence of enzymes being able to penetrate larger pores within the structure. Thus, scaffold destruction will occur throughout the whole structure and not only at the outer surface of the matrix [38,39].

It should be noted that an overwhelming number of studies described in the literature concern analyses of the biodegradation of scaffolds that are not populated with cells [40,41,42,43,44]. However, scaffolds are generally intended to be colonized with cells for clinical purposes. There is no doubt that the presence of cells will alter the scaffold properties, through changing the pattern of the microenvironment [45,46]. How will these changes affect the scaffold biodegradation? There is practically no information on this in the literature.

Thus, the study of scaffold biodegradation is a very important issue that requires detailed analysis. Studies of such biodegradation at the stage of development and preclinical investigations in vitro will help to establish the specific properties of scaffold degradation, to assess the need for modifications in accordance with the intended purpose, and to predict the behavior of the scaffold in vivo.

This work is devoted to an analysis of the in vitro biodegradation of hybrid hydrogel scaffolds based on the cryoprecipitate of blood plasma and collagen. The subjects of the study were two types of scaffolds differing in the type (origin) of the collagen included in their composition. In order to identify changes associated with the inclusion of cells in such scaffolds, we also conducted comparative analyses of the biodegradation of acellular scaffolds and scaffolds with encapsulated mesenchymal stem cells at different stages of cultivation.

## 2. Materials and Methods

### 2.1. Scaffold Formation

For scaffold formation we used a cryoprecipitate of blood plasma obtained from healthy humans. The cryoprecipitate was standardized, in respect to the amount of fibrinogen, to a final concentration of 6 g/L [47,48]. Our experiments used a pool of cryoprecipitate obtained from 8 donors, the PEGylation of the protein component of this being carried out with PEG-NHS (Sigma-Aldrich, Taufkirchen, Germany). To the PEGylated cryoprecipitate we added a 2% solution of acetic collagen (bovine collagen—type I bovine collagen (Sigma-Aldrich, Taufkirchen, Germany); fish collagen—type I cod collagen, isolated from cod skin [49]) that had been previously neutralized with sodium hydroxide. This composite was injected with a cell suspension in phosphate buffer to result in a cell concentration in the composite of 1.2 × 10^5^ cells per mL. In the case of acellular scaffolds, the cell suspension was replaced with an equal volume of PBS. Then, the composite was transferred into a mold. To polymerize the composite, a thrombin–calcium mixture was then introduced into it: 80 IU/mL of human thrombin (Sigma-Aldrich, Taufkirchen, Germany) in a 1% solution of CaCl_2_. The ratios of the components in the composites were—PEGylated cryoprecipitate: neutralized collagen: PBS/cell suspension in PBS 4.31:2.71:1, respectively, while the ratio of composite to thrombin–calcium mixture was 7.39:1.

The resulting mixtures were kept in their molds for 20 min at a temperature of +22–25 °C. During this period, the scaffolds formed, after which each was transferred to a plastic Petri dish of a larger diameter than the prepared scaffold. (The dimensions of the scaffolds used in our experiments were: diameter 3.2 cm, thickness 0.2 cm). The Petri dishes containing scaffolds were transferred to a CO_2_ incubator at 37 °C, with a humid atmosphere and 5% CO_2_ content. Immediately after their formation, the cell scaffolds were flooded with complete growth medium (culture medium α-MEM, 20% FBS, glutamine (324 µg/mL), with the antibiotics penicillin (33.3 µg/mL) and streptomycin (55.5 µg/mL) (LLC PanEco, Moscow, Russia)) and cultured with a change of growth medium twice a week.

The experimental studies were conducted with 6 types of hybrid fibrin–collagen scaffolds that differed in either the source of the collagen included in the composition (bovine collagen; fish collagen) or in the availability of ASCs and in the duration of their pre-cultivation (Table 1): Type 1—acellular scaffolds with bovine collagen; Type 2—acellular scaffolds with fish collagen; Type 3—scaffolds with bovine collagen and encapsulated ASCs, with the scaffolds being pre-cultivated for 24 h; Type 4—scaffolds with fish collagen and encapsulated ASCs, pre-cultivated for 24 h; Type 5—scaffolds with bovine collagen and encapsulated ASCs with the scaffolds being pre-cultivated for 6 days; Type 6—scaffolds with fish collagen and encapsulated ASCs, pre-cultivated for 6 days.

### 2.2. Adipose Tissue Stem Cell Cultures (ASCs)

We used Subculture 3 adipose tissue stem cells (ASCs). The cells met the criteria defined by the Mesenchymal and Tissue Stem Cell Committee (MSC) of the International Society for Cellular Therapy [50]. The cell phenotype corresponded to the MSC phenotype: the cells expressed CD90+, CD105+, CD73+, and CD44+ and did not express CD45−, CD14−, CD34−, or HLA DR−. The cells provided for 3-way differentiation: Adipogenic, osteogenic, and chondrogenic. Cell viability was 98 to 99%.

The cells were recovered from the adipose tissue of healthy donors, obtained during planned cosmetic operations at the Federal State Budgetary Educational Institution of Higher Education, the Privolzhsky Research Medical University of the Ministry of Health of the Russian Federation (FSBEI HE PRMU MOH Russia). All donors underwent a standard examination and provided voluntary informed consent for the collection and use of their biomaterial for scientific purposes. The ASCs were recovered by thermal fermentation, using Type I collagenase. The cells were cultivated in complete α-MEM growth medium (this complete growth medium contains the following: glutamine, antibiotics, and 20% fetal bovine serum (FBS)) in a CO_2_ incubator (under the following conditions: +37 °C; 5% CO_2_; absolute humidity).

### 2.3. Scaffold Biodegradation Analysis

Analysis of the scaffold biodegradation was conducted in two types of solution: phosphate buffer (PBS)—for passive biodegradation—and a 0.25% trypsin in Versene solution (PanEco, Moscow, Russia)—for enzymatic biodegradation. The analysis was performed using scaffold samples with a diameter of 8 mm (7 samples of each type of scaffold, as specified in Section 2.1). The samples were placed in 24-well plates. To each sample was added 1 mL of PBS or trypsin solution. The plates were kept under standard conditions in a CO_2_-incubator (under the following conditions: +37 °C; 5% CO_2_; absolute humidity) throughout the experiment. At test points (from 2 to 1008 h), 100 μL samples were taken from the wells containing the materials under study. The samples were frozen and stored at 80 °C. The volumes taken as samples were compensated by adding appropriate medium (100 μL) to the corresponding wells [51].

After the scaffold incubation had been completed, the frozen samples were examined. The amount of total protein in the samples was estimated. For this, the samples were thawed (at room temperature; 24 h) and the protein concentration determined using an IRF-456 refractometer (KARAT MT, Moscow, Russia). A calibration graph made using human serum albumin was used to determine the total protein concentration. The determination error rate was 0.02%.

### 2.4. Analysis of Scaffold Structure Densities

Analysis of each scaffold structure density was conducted using a technique that had previously been developed by the authors [52]. The analysis was performed using 3 samples of each of the Type 1 and 2 scaffolds (specified in Section 2.1) and of the scaffolds with encapsulated ASCs cultivated for 10 days under standard conditions in a CO_2_-incubator (under the following conditions: +37 °C; 5% CO_2_; absolute humidity). In order to conduct the analysis, a template (a hollow cylinder allowing the removal of a scaffold fragment with a diameter of 0.64 cm^2^) was used to separate a sample fragment from each cultivated cell scaffold at test points (1, 3, 6, and 10 days). These samples were examined using electron transmission microscopy with a Morgagni 268D microscope (FEI, Hillsboro, OR, USA). The resulting photomicrographs (20 photomicrographs for each type of scaffold at each test point; at 14,000× magnification) were processed using ImageJ software (version 1.50i). The analysis included a threshold binarization procedure. The image field was taken as 100%. The area of interest (the biopolymer part of the structure) and the background image (pore lumen) were selected at the image scanning stage. The percentages of the biopolymer part of the scaffold and of the pore lumen in the scaffold structure were assessed.

### 2.5. Microscopy

The state of the cells in those scaffolds with ASCs was monitored by means of an inverted microscope, a Leica DMI 3000 B with LAS software. V. 3.4 (Leica Microsystems, Wetzlar, Germany). We used both bright field and phase contrast modes.

### 2.6. Statistical Analysis

Statistical analysis was performed using the STATISTICA 6.0 software system (Dell Technologies Inc., Round Rock, TX, USA). Nonparametric statistics techniques were applied, including the Mann–Whitney test and Wilcoxon pairwise comparisons.

## 3. Results

**Figure 1:**
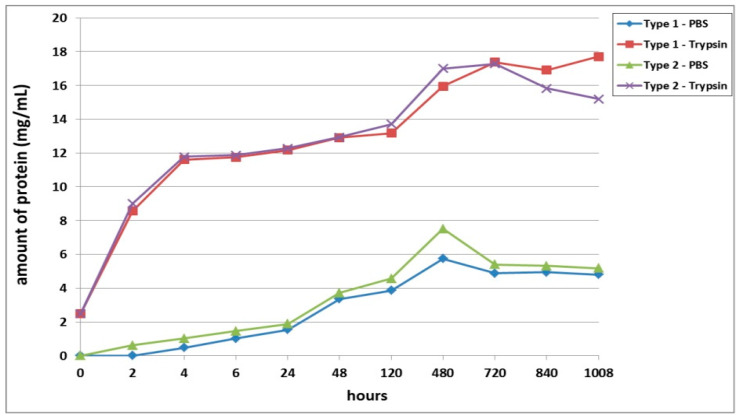
Passive and enzymatic biodegradation of cell-free scaffolds. Note: PBS—passive biodegradation of scaffolds in PBS solution; Trypsin—enzymatic biodegradation of scaffolds in a solution of the hydrolytic enzyme trypsin.

The first series of experimental procedures investigated the passive and enzymatic biodegradation of acellular scaffolds. It was found that passive degradation in the PBS solution proceeded much more slowly than in the trypsin solution (Figure 1). For instance, even after 2 h, the total free protein was determined in the trypsin samples at concentrations of over 8 mg/mL, while the concentration of total protein in samples from scaffolds with bovine collagen (Type 1) with PBS did not exceed 1 mg, and in those from scaffolds with fish collagen (Type 2), no total protein could be detected. However, after 4 h of incubation in PBS, the total protein began to increase rapidly in samples from both types of scaffolds. The peak protein concentrations for the passive degradation (in PBS) of the scaffold samples were observed on Day 20 (480 h) after the start of incubation. It should be noted that the passive biodegradation of the Type 2 scaffolds was marginally faster than that of the Type 1 scaffolds, this being indicated by the higher protein concentrations in the corresponding samples (Figure 1).

The biodegradation of scaffolds in trypsin solution was much more rapid. Both types of scaffolds degraded at almost equal rates up to 5 days of incubation. On Day 20 of incubation (480 h), the protein concentration in the Type 2 scaffold samples was 6.5% higher than that in the Type 1 scaffold samples. As a matter of interest, by Day 30 (720 h), the amount of protein in samples from both types of scaffolds did not differ significantly from a statistical point of view. The maximum values of the protein concentration in Type 1 scaffold samples were recorded on Day 42 of the experiment (1008 h). The pattern of biodegradation effected by the enzyme was significantly different in Type 2 scaffolds during the later stages (840, 1008 h). The analyzed samples demonstrated a decrease in the protein concentration, which indicated either a decrease in the rate of biodegradation of the scaffolds or its termination. To clarify, according to the technique used, after sampling (100 μL), the amount of liquid in the well with the incubated sample was replenished with the same volume of liquid, that is, the solutions were effectively diluted. Thus, if biodegradation had significantly slowed down or stopped, the amount of protein in the samples would have decreased, while if the scaffold biodegradation had continued, the amount of protein in the samples would likely have appeared to remain constant or to increase. Summarizing the results, one can conclude that after 48 h, the biodegradation rate of the Type 2 scaffolds was higher than that of the Type 1 scaffolds. This was indicated by the initial marked increase in protein in the samples (Type 2) followed by its subsequent decrease.

**Figure 2:**
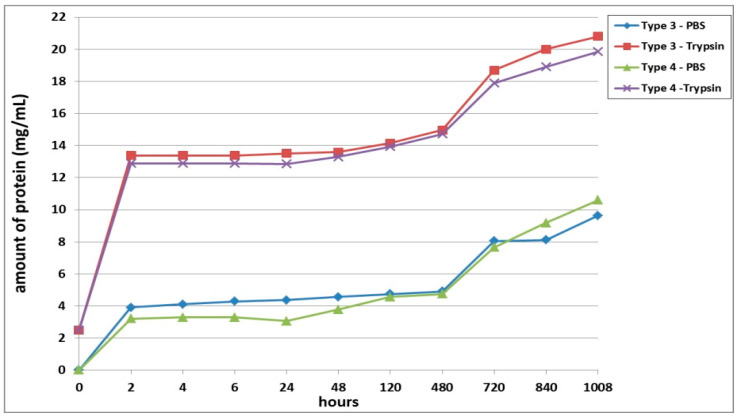
Biodegradation of scaffolds with encapsulated ASCs pre-cultivated for 24 h prior the analysis. Note: PBS—passive biodegradation of scaffolds in PBS solution; Trypsin—enzymatic biodegradation of scaffolds in a solution of the hydrolytic enzyme trypsin.

The second series of experiments involved an analysis of the biodegradation of scaffolds with encapsulated ASCs pre-cultivated for 24 h before being transferred to the biodegradation experiment. As in the first series, proteolytic degradation of the scaffolds (in trypsin solution) proceeded much faster than did the passive biodegradation in PBS solution (Figure 2). The passive biodegradation of the scaffolds was characterized by an increase in protein concentration in the samples from both types of scaffolds over the period of the whole experiment. The protein concentration gradually increased over the period from 2 h to Day 20 (480 h). During this period, the Type 3 scaffolds degraded more actively, which was indicated by the higher protein concentration in these samples compared to the Type 4 scaffold samples. Furthermore, there was a sharp increase in protein in the samples, which indicated an acceleration of the degradation process. It should be noted, however, that the amount of protein in the Type 4 scaffold samples still exceeded the amount of protein in the Type 3 samples by the end of the experiment (840 h and 1008 h, respectively). Thus, the relative rates and nature of the passive biodegradation of the scaffolds appears to have changed during these later stages.

The biodegradation of the scaffolds in trypsin solution proceeded much more rapidly compared to the passive biodegradation. For instance, even after only 2 h of incubation, the samples from both scaffold types showed significantly high protein concentrations (Figure 2). Throughout almost the entire analysis period, the enzymatic biodegradation of the Type 3 scaffolds was more pronounced compared to that of the corresponding Type 4 scaffolds. The increase in protein concentration in the Type 3 scaffold samples during the first 2 days was, on average 3.4% (significantly) higher than the values in the Type 4 scaffold samples. However, in samples taken at 120 h and 480 h, the values of the total protein concentration did not show statistically significant differences between the two types of scaffolds. After 20 days (480 h), the situation then completely changed. On Day 30 of the experiment, statistically significant differences could again be detected for the protein concentrations in samples from Type 3 scaffolds and Type 4 scaffolds, and this situation continued until the end of the experiment. As at the early stages, the Type 3 scaffolds appeared to be degraded more rapidly than the Type 4 scaffolds during this period (720 to 1008 h). Between Day 20 and Day 30, an abrupt increase in protein concentration was seen for both types of scaffolds. For instance, on Day 30, the protein concentration in the Type 3 scaffold samples increased by 20%, and in the Type 4 scaffold samples, by 18%, compared to the values registered on Day 20 (480 h). Subsequently, and until the end of the experiment, the protein concentration in the samples from both types of scaffolds increased, indicating an ongoing process of biodegradation.

Summarizing the results obtained, it can be concluded that the Type 3 scaffolds degraded to a greater extent than Type 4, both in the enzyme and in the PBS solutions. However, at a later stage, the pattern of biodegradation in the PBS solution changed, and the Type 3 scaffolds showed greater resistance to degradation than did the Type 4 scaffolds. The protein concentrations in samples of both types of scaffolds in both types of solution continued to increase until the end of the experiment, indicating that the process was ongoing and that the scaffolds continued to degrade.

**Figure 3:**
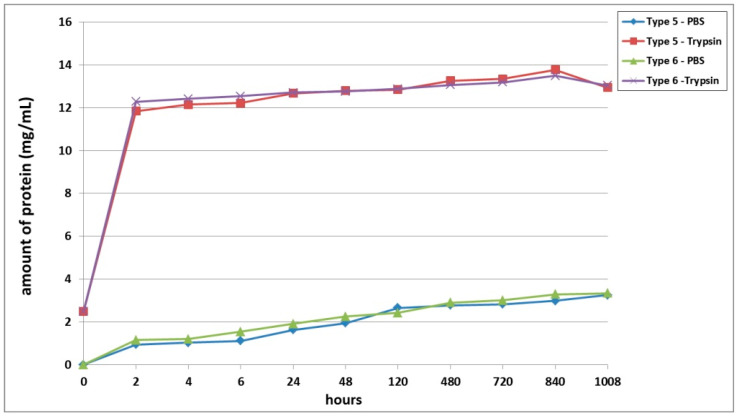
Biodegradation of scaffolds with encapsulated ASCs, pre-cultivated for 6 days before the analysis. Note: PBS—passive biodegradation of scaffolds in PBS solution; Trypsin—enzymatic biodegradation of scaffolds in a solution of the hydrolytic enzyme trypsin.

A third series of experiment was dedicated to studying the biodegradation of scaffolds with encapsulated ASCs, pre-cultivated for 6 days before being subjected to biodegradation. It was shown that, as with the series 1 and series 2 experiments, the scaffold biodegradation in the enzyme solution proceeded much more rapidly than in the PBS solution (Figure 3). During the analysis of passive degradation, even just 2 h after the start of incubation, protein was recorded in samples from both types of scaffolds. However, the concentration of total protein in the Type 6 scaffold samples was 18.5% higher than that in the Type 5 scaffold samples. Over the course of incubation and until the end of the experiment, the protein concentration in samples from both types of scaffolds gradually increased. Throughout practically the entire experiment, the degree of passive degradation of the Type 6 scaffolds was higher than that of the Type 5 scaffolds. It should be noted that the differences in biodegradation between the two types of scaffolds were more pronounced during the early stages compared to the later periods. At the end of the experiment, on Day 42 (1008 h), the protein concentration in the samples was the same for both types of scaffolds. The absence of a plateau or a decrease in protein concentration in the samples indicated that the biodegradation had continued and that neither type of scaffold had been completely degraded by the end of the experiment (1008 h).

During the analysis of the enzymatic biodegradation results, it was found that 2 h after the start of the experiment, significant protein concentrations could be observed in samples from both types of scaffolds—over 11.5 mg/mL. As with the results of passive biodegradation, the amount of protein in the Type 6 scaffold samples exceeded the values from the Type 5 scaffolds. However, this difference was much smaller and did not exceed 4%. Progressively during the experiment, the protein concentration in samples from both types of scaffolds gradually increased. In the early stages (up to 24 h), the relative differences between the samples from the Type 5 and Type 6 scaffolds remained. Then, during the period from 24 h to Day 20 (480 h), the protein concentration in the samples from both types of scaffolds became equivalent. After 480 h, the biodegradation of the Type 5 scaffolds proceeded marginally faster. However, these later differences were in the range of only 1.5 to 2.1%. At the final point of the experiment (1008 h), the protein concentration in both types of scaffolds decreased, indicating a slowdown or termination of the biodegradation process.

**Figure 4:**
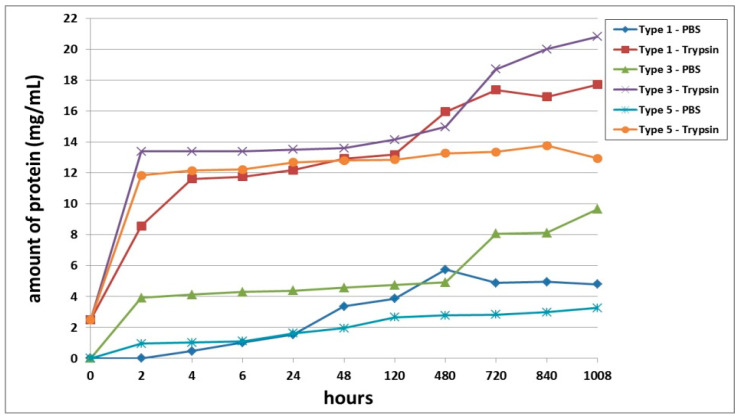
Comparative analysis of passive and enzymatic biodegradation of acellular scaffolds made using bovine collagen (Type 1) and of scaffolds with encapsulated ASCs, pre-cultivated for 24 h (Type 3) or for 6 days (Type 5). Note: PBS—passive biodegradation of scaffolds in PBS solution; Trypsin—enzymatic biodegradation of scaffolds in a solution of the hydrolytic enzyme trypsin.

Furthermore, we conducted a comparative analysis of the biodegradation of scaffolds with bovine collagen—acellular (Type 1) or with encapsulated ASCs pre-cultivated for 24 h (Type 3) and for 6 days (Type 5). It was found that the most rapid degradation was seen for scaffolds with encapsulated ASCs, pre-cultivated for 24 h (Figure 4). This was the case for both passive and enzymatic degradation. Scaffolds pre-cultivated for 6 days with encapsulated ASCs had the lowest degree of biodegradation in both types of solution. Acellular scaffolds (Type 1) occupied an intermediate position between the scaffolds with encapsulated ASCs (Types 3 and 5). It should be noted that during the early stages of observation (Days 1–2), the picture was marginally different. During this period, the Type 1 scaffolds showed a lower biodegradation rate compared to the Type 5 scaffolds. This was typical for both passive and enzymatic degradation.

**Figure 5:**
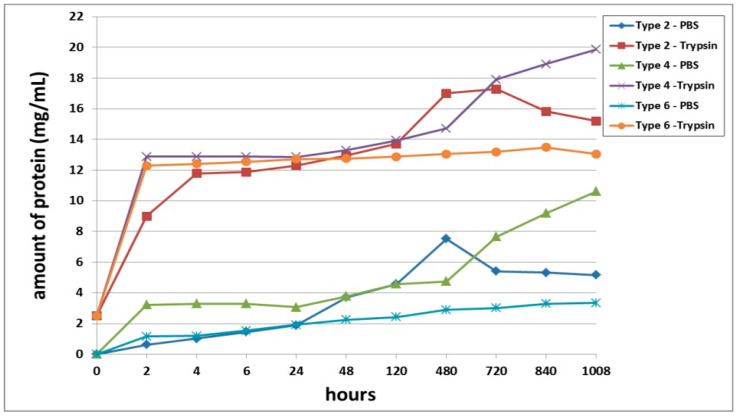
Comparative analysis of passive and enzymatic biodegradation of acellular scaffolds with fish collagen (Type 2), and scaffolds with encapsulated ASCs, pre-cultivated for 24 h (Type 4) or for 6 days (Type 6). Note: PBS—passive biodegradation of scaffolds in PBS solution; Trypsin—enzymatic biodegradation of scaffolds in a solution of the hydrolytic enzyme trypsin.

A comparative analysis of the biodegradation of scaffolds with fish collagen—acellular (Type 2), with encapsulated ASCs, pre-cultivated for 24 h (Type 4) or for 6 days (Type 6)—demonstrated dynamics similar to the biodegradation of the scaffolds made with bovine collagen. For instance, during the experiment, the Type 4 scaffolds degraded the fastest. This was typical for both types of degradation (passive and enzymatic). Despite similar general dynamics (compared to the bovine collagen scaffolds) there were some differences in the process of biodegradation of the fish collagen scaffolds. During the very early stages (2–4 h), the passive biodegradation of the Type 2 scaffolds proceeded at the slowest rate. During the period from 6–24 h, the biodegradation of the Type 2 and Type 6 scaffolds in the PBS solution proceeded at corresponding rates. Starting from 48 h, the picture changed significantly. The protein concentration in the Type 6 scaffold samples continued to increase smoothly and reached a plateau by the end of the experiment. Meanwhile, the protein concentration in the Type 2 scaffold samples abruptly increased and reached a peak by Day 20 (480 h). Then, there was a decline. Thus, at a later stage, the Type 2 scaffolds degraded much more rapidly compared to the Type 6 scaffolds (Figure 5). During this period, the situation was similar to the passive biodegradation of the Type 1 and Type 5 scaffolds (Figure 4). The enzymatic degradation of the Type 6 scaffolds in the early stages proceeded much more quickly than the degradation of the Type 2 scaffolds and approached the rate of degradation observed for the Type 4 scaffolds. However, after 48 h, the situation changed and the rate of degradation of Type 2 scaffolds abruptly increased and was significantly higher than that of the corresponding Type 6 scaffolds until the end of the experiment.

**Figure 6:**
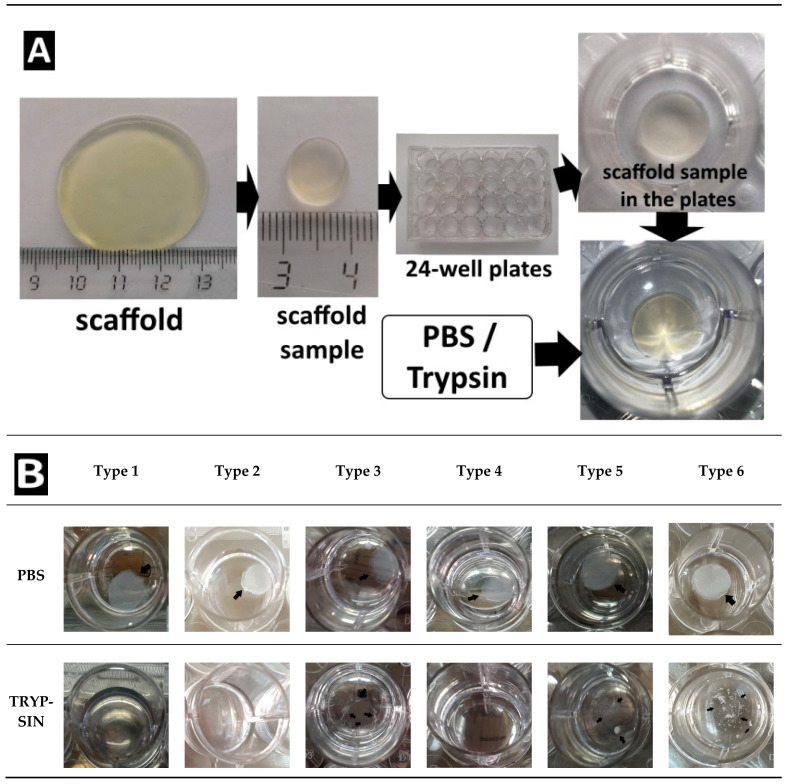
(**A**) Introduction to the experiment. Representative photographs of the scaffolds. The appearance of the scaffolds of all Types before their introduction into the experiment was identical. (**B**) Appearance of scaffold samples after 1008 h of biodegradation in vitro.

Figure 6 shows a scaffold before its introduction into the experiment (Figure 6A) and views of the scaffold samples after 1008 h of treatment (Figure 6B). When analyzing the photographs, it can be seen that the scaffolds in the process of passive degradation, regardless of their Type, retained their shape and were well visualized. A different picture was observed when analyzing photographs of scaffolds being degraded by trypsin. The Type 1 and Type 2 complete scaffolds could no longer be visualized. In such wells with the Type 1 scaffolds, subtle whitish microfragments of the scaffold could be detected. Some wells with Type 3 scaffolds showed small fragments of the scaffold, although in others they were missing. No scaffold residues could be observed in the wells of the plate with the Type 4 scaffolds, the liquid was transparent. In the wells with the Type 5 and Type 6 scaffolds, residual fragments of the scaffolds could still be easily visualized, which indicated that the degradation process was incomplete.

**Figure 7:**
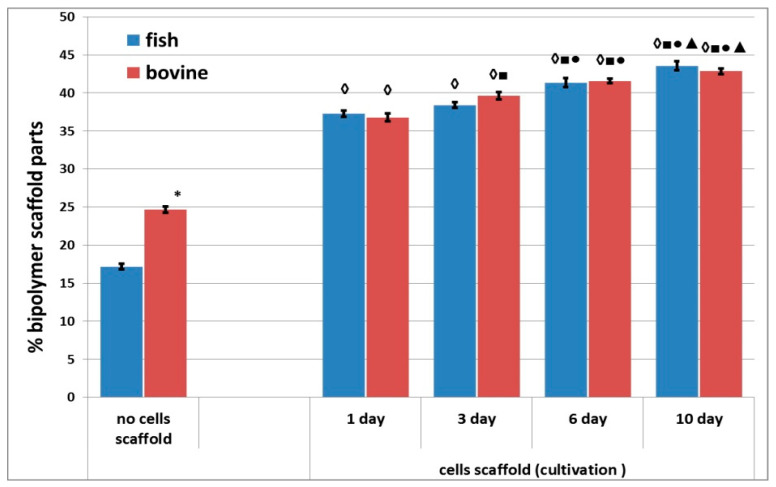
Density of the structure of acellular and cellular scaffolds during cultivation. Note: “Fish” means scaffolds with fish collagen, “bovine”—scaffolds with bovine collagen.∗—*p* < 0.05 comparison of acellular bovine collagen scaffolds with acellular fish collagen scaffolds; ◊—*p* < 0.05 comparison of acellular scaffolds; ■—*p* < 0.05 comparison with Day 1; ●—*p* < 0.05 comparison with Day 3; ▲—*p* < 0.05 comparison with Day 6.

A comparative study of the densities of the structures of the acellular scaffolds was conducted. The structure of the acellular scaffolds with bovine collagen was denser than those made with fish collagen. For instance, the percentage of the biopolymer part of the scaffolds with bovine collagen was almost 1.5 times greater than in the corresponding scaffolds with fish collagen (Figure 7). Furthermore, an analysis was conducted of the changes occurring in the density of the structure of cell scaffolds during cultivation. It was shown that the percentage of the biopolymer part increased in scaffolds with either type of collagen during periods of cultivation ranging from 1 to 10 days. Thus, the structure of the scaffolds became denser in the course of cultivation. It should be noted that when comparing the results for Day 1 and Day 3 for scaffolds with fish collagen, only a slight increase in the percentage of the biopolymer part of the scaffold was observed. By contrast, statistically significant changes occurred in the bovine collagen scaffolds. However, there were no statistically significant differences found between the scaffolds with fish and bovine collagen when compared at the same cultivation points, for example, on Day 1 or Day 10. As a matter of interest, significant differences were revealed when comparing the density of the acellular and cellular scaffold structures cultivated for 24 h. At this time, the percentage of the biopolymer portion of the cellular scaffolds with either type of collagen was significantly higher than that of the acellular ones (Figure 7).

## 4. Discussion

Scaffolds within each series of experiments were formed by using the same technique, maintained under the same conditions, and differing only in the type of collagen used in their construction (Series 1: Type 1—bovine collagen; Type 2—fish collagen; Series 2: Type 3—bovine collagen; Type 4—fish collagen; Series 3: Type 5—bovine collagen; Type 6—fish collagen). The results revealed differences in the rates of biodegradation between the types of scaffolds under study (Figure 1, Figure 2 and Figure 3) in each series. Indeed, this applied to both passive and enzymatic biodegradation. Thus, it can be concluded that the type of collagen in hybrid hydrogel scaffolds can influence specific aspects of their biodegradation. Previously, we had demonstrated that acellular hybrid hydrogel scaffolds formed using different types of collagen differed in their structural characteristics and elastic properties [48]. For instance, acellular bovine collagen scaffolds are characterized by a denser structure and greater elasticity compared to fish collagen scaffolds. The scaffolds were formed under enzymatic hydrolysis, thrombin being added to the starting composite in order to deconjugate the polypeptide bonds in the main structure-forming proteins—the fibrin and collagen. As this results in the formation of products of enzymatic hydrolysis of the collagen and fibrinogen [53,54], the scaffold structure is formed from these. It has been shown that Type 1 collagens of different species origin differ in molecular weight and that this determines the differences in the products of these protein hydrolyses and, ultimately, therefore, the differences in the structure of the resulting scaffolds [55,56]. It is predictable that such differences in structural characteristics would be a factor involved in determining the differences in the rate of scaffold biodegradation. This was confirmed by the data obtained in our first series of experiments. Acellular scaffolds with a denser structure (bovine collagen) degraded less quickly than those scaffolds that had a lower density structure (those with fish collagen).

Differences between passive biodegradation and enzymatic biodegradation are to be expected. The composition of the phosphate buffer is close to the water–salt phase of human blood; therefore, passive biodegradation proceeded in the absence of substances capable of causing the active decomposition of the scaffold structure. Enzymatic biodegradation proceeded under the action of trypsin, an enzyme of the class of hydrolases that breaks down peptides and proteins. The investigated scaffolds have a protein nature, so it is not surprising that under the action of this enzyme they degraded more actively than without it.

Moreover, it is possible that differences in the ratio of amino-acid residues comprising the different collagens could also affect the scaffolds’ enzymatic biodegradation properties. It is known that trypsin targets diaminomonocarboxylic acids such as lysine, arginine, and histidine. Bovine collagen contains less lysine and arginine but a lot of histidine, while the composition of fish collagen is the opposite [57,58,59]. Therefore, it is likely that the collagen hydrolysates involved in scaffold formation also contain different amounts of “target” acids. The latter would be expected to contribute significantly to the biodegradation properties of the scaffolds when attacked by enzymes and could therefore determine the resulting degradation rate differences between scaffolds with bovine or fish collagen.

There is no doubt that the revealed differences in the biodegradation of acellular and cellular scaffolds are associated with the introduction of a cellular component into the scaffold. However, after different periods of cultivation, the effects of the presence of such cells varied. For instance, scaffolds pre-cultivated for 24 h were more vulnerable to degradation than were scaffolds either without cells or those that had been pre-cultivated with cells for 6 days. What could be the explanation? The cellular component is introduced into the composition of the composite during scaffold formation. The polymerization of the structure-forming proteins occurs only at the next stage [60]. That is how the cells become “built-in” to the scaffold structure. The formation of the scaffold occurs mainly due to the development of covalent bonds between the hydrolysates of the structure-forming proteins. It is unlikely that the connection between cells and the scaffold’s structural elements are formed by covalent bonds. At this stage (the first day after formation), one can propose several mechanisms of cell “retention” in the scaffold structure: becoming mechanically fixed between the scaffold’s structural elements, adsorption, ionic interactions (the bilipid membrane of cells is negatively charged, whereas the scaffold protein structure may have areas with a positive charge), etc. All these types of bonds are fairly weak compared to covalent bonds. It should be noted that as the cells occupy additional space inside the scaffold, they will therefore prohibit the formation of covalent bonds between its structure-forming elements in these areas. M. Mazzeo [61] demonstrated that, in the case of cells encapsulated in a synthetic hydrogel during the formation of its structure, the proportion of covalent bonds was decreased in comparison with similar acellular scaffolds. It has been estimated that a loss of up to 80% of cross-bonds is possible in the case of cell encapsulation in a hydrogel [62]. This provides for the formation of “weakened” areas in the scaffold structure in those places where the cells are “built-in”. Thus, the scaffold structure is weakened, making it more vulnerable to biodegradation than are acellular scaffolds. Moreover, the literature indicates that during the process of incorporation into the scaffold structure, the cells can leave target areas exposed to subsequent enzyme attack, and therefore, such a scaffold becomes more vulnerable to aggressive action by enzymes [61].

**Figure 8:**
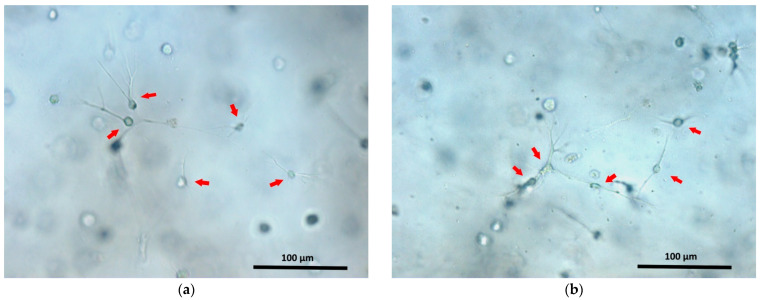
ASCs cultivated for 24 h in fish (**a**) and bovine (**b**) collagen scaffolds. The arrows indicate some of the ASCs. Cellular processes are well visualized in adhered ASCs.

It is also interesting that the structure of acellular scaffolds was less dense than the structure of cell scaffolds cultivated for 24 h. However, the differences in structural density observed for acellular scaffolds with fish and bovine collagen were reduced when the scaffolds contained cells. Why does the scaffold structure change when cells are encapsulated in it? We cannot yet provide a complete answer to this question. However, one may conjecture as follows. The protein hydrolysis and subsequent self-assembly of the structure from the hydrolysate elements are ionic processes. It is known that the cell membrane is negatively charged [63]. Thus, it is likely that the participation in the structure of the charged cells affects the ionic processes occurring during scaffold formation. As a result, the structures of acellular scaffolds and of scaffolds with a cellular component would be different. This issue definitely requires further investigation. The activity of cells related to adhesion and to the contraction of the hydrogel’s soft structure during the formation of projections by cells can also contribute to the process of induration of the scaffold structure. One can see that after 24 h, the cells have adhered to the structural elements of the scaffold and have sent out cellular projections (Figure 8). However, it is unlikely that such cellular processes make a significant contribution to the induration of the scaffold structure during the first 24 h of cultivation. That 24 h is more a period of initial cell adaptation to their new environment, being too short for the cells to significantly transform the scaffold structure as a result of their vital activity.

The assessment of the biodegradation of cellular scaffolds pre-cultivated for 6 days showed that they were the least vulnerable to degradation. These scaffolds were more resistant to biodegradation than were either acellular scaffolds or scaffolds pre-cultivated for 24 h. There is no doubt that an increase in the resistance of scaffolds to biodegradation is related to the vital activity of any cells they contain. We have previously shown that ASCs cultivated in scaffolds proliferate, demonstrate three-dimensional growth, and form a developed cellular network [47,48]. It is known that during the formation of such a cellular network, the cells build strong intercellular connections and can cause the contraction of the scaffold structure [64]. One can assume that the formation of the cellular network could increase the scaffold’s resistance to biodegradation. A second factor helping to increase stability may be a change in the structure of the scaffold. It is known that stem cells can secrete various biologically active substances, for example, cytokines, growth factors, and various inhibitors [46,65,66,67]. Our studies have demonstrated that ASCs cultivated in the scaffolds under analysis maintain their secretory activity [47,68]. Moreover, cells in the scaffold can both produce and structure collagen [65,69]. Ultimately, the cells dynamically remodel the scaffold structure [70]. We demonstrated that during the period from Day 1 to Day 10, the structure of the scaffolds under analysis become significantly denser. Thus, during a long-term pre-cultivation (6 days), the cells presumably remodel the scaffold structure, resulting in an increase in its resistance to biodegradation, both in comparison with acellular scaffolds and with cellular scaffolds pre-cultivated for only 24 h.

Based on the results obtained, we feel that the properties of the scaffolds represented in this work will make them valuable for the restoration of soft tissue defects, for example, of the skin. It is known that the regenerative process of most soft tissues involves several stages, including hemostasis, inflammation, proliferation, and remodeling. However, there are no clear boundaries between these stages. Furthermore, starting from the stage of inflammation and ending with the complete remodeling of the tissue, the process of angiogenesis proceeds simultaneously. The process of complete tissue recovery depends on the severity of the injury and can take from a month to a year [71]. Nevertheless, given that one of the main functions of scaffolds is to support cells, it is important that special attention should be paid to the stage of cell proliferation. During this period, cells are actively dividing and migrating into the scaffold structure and producing the necessary substances that ensure the process of recruiting further cells from surrounding tissues to contribute to the repair of the damaged tissue. The duration of active proliferation ranges from several weeks to a month [72]. It is especially during this period that the cells need support in the form of a scaffold. It must ensure both the adhesion of the cells and their continued vital activity, without impeding the functional activity of those cells. Thus, the overall time for degradation of scaffolds intended for the restoration of soft tissues should be at least 4 weeks, as this will allow for the maintenance of the active process of regeneration of the damaged tissues. For example, in the work of X. Zhou (2019), the biodegradation of several types of scaffolds based on polycaprolactone and polydioxanone (a rapidly decomposing polyester) was studied. The degradation of the scaffolds in vitro took place in a PBS solution for 12 weeks. After this time, the remaining mass of the polydioxanone-based scaffold was only 17.19% of the initial mass of the construct. When studying degradation in vivo, the authors showed that by 4 weeks after implantation, polydioxanone-based scaffolds had already lost their fibrous structure. This difference is associated with the activity of enzymes, macrophages, and other factors in the lesion. The authors concluded that the results obtained support the use of such scaffolds for the restoration of tissues that do not require a long healing period [39]. In the work of R. Nazir, scaffolds developed for the restoration of heart valve defects were investigated. The authors showed that composite scaffolds based on collagen and hydroxyapatite can retain their structure for up to 28 days under conditions of passive degradation in vitro but rapidly (within 35 h) degrade in the presence of enzymes [73]. In the work of G. Ramanathan et al. (2017), a study of hybrid collagen scaffolds for the replacement of skin defects was conducted [74]. Under the conditions of enzymatic hydrolysis in vitro, these scaffolds lost up to 60% of their weight within 24 h. However, despite such rapid degradation in vitro, they provided for a regenerative process of restoring a skin-wound defect in rats in vivo for 12 days. V. Sharma et al. (2016) conducted a study of the biodegradation of well-known commercial products: Integra—a collagen-based scaffold—and SmartMatrix—a fibrin-based scaffold [51]. It was shown that, in vitro, under the action of a 0.25% solution of trypsin, the fibrin-based scaffold completely degraded within 42 days (1008 h). Under the same conditions, the collagen-based scaffold did not completely degrade—residual fragments of the scaffold could still be visualized, and the protein concentration in the samples continued to increase throughout the experiment. Integra and SmartMatrix are well-established as scaffolds intended for the repair of skin-wounds, including burn wounds. The above examples give reason to believe that the scaffolds presented in our work, which have a fibrin–collagen nature and degrade in the presence of a hydrolytic enzyme over a period lasting at least 4 weeks, should also be useful in repairing wound defects of the skin or other soft tissues. While their relatively high rate of biodegradation makes them ineffective for treating injuries that require a longer recovery period, such as extensive bone defects, the scaffolds we have described do provide a good regenerative potential for relatively quickly recovering soft tissues, since their timely degradation will avoid the development of scars and chronic inflammation in the lesions.

Revealing the distinctive features of scaffold biodegradation, with their dependence on the type of collagen and on the presence of a cellular component, provides us with the information needed to predetermine the progress of scaffold biodegradation. This makes it possible to choose scaffolds with the necessary characteristics to ensure the efficient regeneration of individual types of tissue with particular types of damage. It should be emphasized that the biodegradation of the scaffold material should always be chosen to correspond to the specific clinical situation. Of course, in order to carry out such a selection, it is necessary to conduct in vivo studies on experimental animals with particular damage models. This is the subject of our further research. However, based on the studies we have conducted, it is already clear that the nature of scaffold biodegradation can be determined by the choice of scaffold components and that it very much depends not only on the presence of a cellular component but also on the stage of cultivation. Thus, the data presented in this paper should be useful in the development of scaffolds for various applications, as they give an idea of the influence of the above factors on the nature of biodegradation. The latter allows one to predict the properties of scaffolds at the development stage and to choose appropriate directions of modification. We hope that this data will be useful to researchers working in the field of scaffold technologies and tissue engineering.

## 5. Conclusions

We analyzed the biodegradation of hybrid fibrin–collagen acellular scaffolds and scaffolds with encapsulated ASCs at various stages of cultivation. It was expected that the enzymatic degradation of all the types of scaffolds under analysis would proceed much more actively than passive degradation in PBS solution. It was shown that scaffolds differing only in the type of collagen in their composition (fish or bovine) degrade at different rates. A comparative study of acellular scaffolds and cellular scaffolds demonstrated that scaffolds with encapsulated ASCs cultivated for 24 h were the most vulnerable to degradation. By contrast, scaffolds with ASCs pre-cultivated for 6 days had the highest resistance to degradation. Summarizing the results obtained, one can conclude that the type of collagen comprising part of hybrid scaffolds can influence their biodegradation properties, while the inclusion of a cellular component in scaffolds significantly changes their structural and biodegradation properties. Depending on the pre-cultivation time, stability of the scaffolds against biodegradation also differed significantly. Based on the results obtained, one can conclude that during the development of scaffolds that include collagen, it is important to take into account not only its type but also the species origin, as that has a significant impact on both the structural characteristics and biodegradation properties of such scaffolds. Considering the major influence that the cellular component can have on scaffold properties, it will be important to conduct research regarding the stage of pre-cultivation at which it is best to implant the scaffold into tissues that are being repaired. This will make it possible to extend the in vitro data to in vivo systems with greater accuracy and to predict the biodegradation properties of a scaffold after implantation into damaged tissues. Thus, although the study of scaffold biodegradation is a complex, multi-stage process, it is a necessary step in order to be able to specify and forecast the properties of scaffolds or tissue-engineered structures at the stage of preclinical studies.

## Figures and Tables

**Table 1:** The types scaffolds.

Type Scaffold	Collagen	ASCs	Pre-Cultivation
Type 1	bovine	-	-
Type 2	fish	-	-
Type 3	bovine	+	24 h
Type 4	fish	+	24 h
Type 5	bovine	+	6 days
Type 6	fish	+	6 days
